# Conservative Management in End-Stage Kidney Disease between the Dialysis Myth and Neglected Evidence-Based Medicine

**DOI:** 10.3390/jcm13010041

**Published:** 2023-12-21

**Authors:** Francesca K. Martino, Giacomo Novara, Federico Nalesso, Lorenzo A. Calò

**Affiliations:** 1 Nephrology, Dialysis, Transplantation Unit, Department of Medicine (DIMED), University of Padova, 35124 Padua, Italy; francesca.martino.k@gmail.com (F.K.M.); federico.nalesso@unipd.it (F.N.); 2 Department of Surgery, Oncology and Gastroenterology, Urology Clinic University of Padua, 35124 Padua, Italy

**Keywords:** elders, comorbid, end-stage kidney disease, conservative management

## Abstract

In the last few decades, the aging of the general population has significantly increased the number of elderly patients with end-stage kidney disease (ESKD) who require renal replacement therapy. ESKD elders are often frail and highly comorbid with social issues and seem to not benefit from dialysis in terms of survival and quality of life. Conservative management (CM) could represent a valid treatment option, allowing them to live for months to years with a modest impact on their habits. Despite these possible advantages, CM remains underused due to the myth of dialysis as the only effective treatment option for all ESKD patients regardless of its impact on quality of life and survival. Both CM and dialysis remain valid alternatives in the management of ESKD. However, assessing comorbidities, disabilities, and social context should drive the choice of the best possible treatment for ESKD, while in elderly patients with short life expectancies, referring them to palliative care seems the most reasonable choice.

## 1. The Elderly Frail Patient with End-Stage Kidney Disease (ESKD) and Their Treatment Options

In the last few decades, in Western countries, population aging has increased the number of elderly patients with end-stage kidney disease (ESKD) who potentially need renal replacement therapy (RRT). In 2019, the Italian Register of Dialysis reported about 500 individuals per million people (pmp) per year aged over 75 had reached ESKD [1]. In 2020, the United States Renal Data System declared an incidence of ESKD in 1447 pmp among individuals aged ≥75 years, about three times the incidence in patients aged 45–64 years (598 pmp) [2]. In 2022, the Danish Civil Registration System reported a prevalence of about 58% for patients over 70 years old with an estimated glomerular filtration rate (eGFR) below 15 mL/min [3]. The same Danish register reported a higher prevalence of diabetes, hypertension, and cardiovascular (CV) disease in ESKD patients over 70 years of age compared to patients of the same age without chronic kidney disease (CKD).

The presence of CKD at stages 4 and 5 and older age increases CV mortality by 2–3 times [4]. Specifically, in patients over 80 years of age with ESKD, life expectancy is three times lower than in their peers with normal renal function [4]. In the CKD population, the traditional risk factors, such as diabetes mellitus, hypertension, congestive heart failure, clinical depression, history of ischemic heart disease, cerebrovascular disease, the presence of cancer, and physical and nutritional impairment, also contribute to increasing the CV mortality risk [5,6]. Thus, elderly patients with ESKD and high comorbidity have an even higher mortality risk [7,8,9]. Finally, some non-traditional risk factors for CV events related to ESKD, such as anemia, phosphate increase [10], and potassium imbalance [11], further aggravate the mortality risk in CKD patients.

In the elderly and frail context, Soucie et al. [12] showed a significant increase in mortality in the first 100 days after RRT beginning in patients >75 years of age, with a mortality rate of about 50% and a five-times higher risk of death after the start of RRT (OR equal to 5.0 [3.2–7.8]). Other studies have evaluated prognosis after RRT begins, showing a significant increase in death risk in the presence of multiple comorbidities. Couchoud et al. detected mortality rates ranging from 8% to 70%, according to the presence of comorbidities [13]. Cohen et al. found older age, dementia, peripheral vascular disease, and decreased albumin to be independent predictors of mortality risk in the first six months after dialysis [14], and Muskulin et al. reported the comorbidities’ severity as a stronger predictor of death in dialysis patients [15].

Further small observational studies have compared the survival between RRT and CM with doubtful results [16,17,18,19]. In 2017, a meta-analysis suggested a significant difference in survival between dialysis and CM patients, reporting median survival estimates of 8–67 and 6–30 months, respectively [20]. However, high heterogeneity among the studies and the significant differences between RRT and CM patients in terms of comorbidities and age precluded definitive conclusions.

The mortality risk stratification in ESKD should also consider socio-economic issues, such as social isolation, family context, type of care, and health care system [21,22]. After 80 years of age, the perception of life and death may be far from reason: the loss of spouses, relatives, and friends, poor social activity, and quality of life may influence treatment choices. Some conditions, such as older age, comorbidities, and disability with the need for a caregiver, seem to increase the likelihood of the free choice of CM [23]. Unfortunately, the patient’s intentions for ESKD treatment are often obscured by the idea of life-saving treatment, while understanding their needs can fortify the therapeutic alliance between the patient and the nephrologist.

CM fits perfectly into ESKD elderly patients with preserved urine output for several reasons:

- CM permits the effective control of uremia [24]. Specifically, a low-protein diet significantly improves urea levels [25], calcium–phosphorus metabolism [26], and metabolic acidosis [27], while it can worsen potassium control. Fortunately, new resins can help to reduce potassium absorption and the risk of hyperkaliemia with few side effects.
- CM slows the progression of eGFR loss [28,29], which already seems slower in elderly patients. Yeh et al. showed a significant risk decrease in ESKD in patients over 65 years of age with moderate to severe CKD [30]. Similarly, Santos et al. detected a decline in lower kidney function before dialysis in patients over 75 years of age [31].
- CM limits their hospitalization, reducing hospital access related to dialysis complications, which are more frequent in older patients [32].
- CM allows them to maintain their habits with slight impairment of quality of life. Some comparative studies between RRT and CM about the perceptions of quality of life in aged patients showed worse scores in RRT patients [33,34,35]. Furthermore, Kurella Tamura et al. reported a substantial and sustained decline in the functional status of frail patients with ESKD after dialysis initiation [36], suggesting a negative influence on the quality of life of dialysis patients regarding the contextual decline in executive function, which limits the ability to manage emotions and actions.

## 2. Choice of Treatment

**Figure 1:**
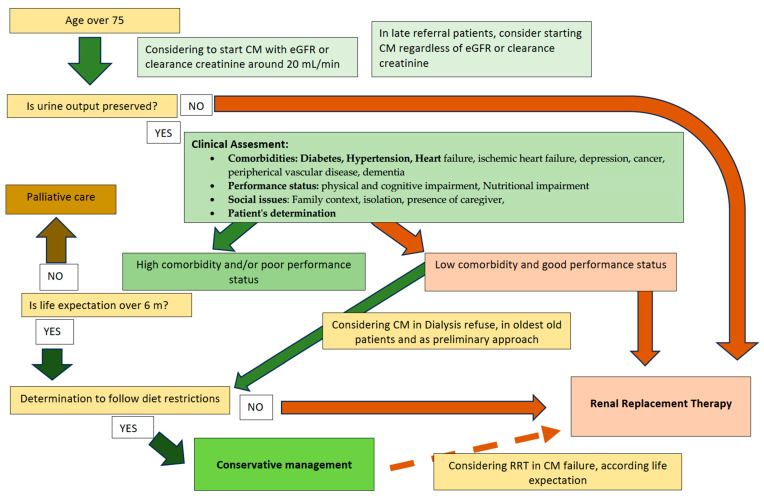
Our suggested flowchart for the conservative management of elderly patients with ESKD. CM conservative management, RRT renal replacement therapy.

Individualizing the best possible treatment (RRT vs. CM) in octogenarian comorbid patients remains challenging for nephrologists [37,38]. Even though, in the last decade, dialysis access has become available to an increasing number of patients, its effects on survival and quality of life in frail and elderly patients seem doubtful and fail to show a clear advantage. Based on the strength of the evidence, we propose a logical approach to manage elderly and not oliguric ESKD patients (Figure 1), considering the following points:

- Survival: the elderly patients should be screened for comorbidities, social status, and performance status [39,40].
- Elderly patients without comorbidities and with a good performance status should receive RRT, peritoneal dialysis, or hemodialysis. However, in this case, it seems reasonable to propose, as a preliminary approach, CM to slow the progression of CKD [41,42,43] and, consequently, delay the need for RRT. We have no evidence that the early beginning of RRT might improve survival [44,45]. Finally, considering the oldest-old ESKD patients with low CV risk and their expectation of years of life, well-managed CM can be a definitive treatment option for most cases.
- Elderly, high-comorbid patients with a poor performance status should receive CM, considering the negative impact of dialysis on survival and quality of life [12,36]. Unless they have a life expectancy under six months, in this case, referring them to palliative care seems the best reasonable choice [46]. Although there are differences between conservative and palliative therapy regarding the type of care provided to the patient and the purpose of treatment, these two approaches are often confused in the nephrology field. In our opinion, this mess explains why conservative therapy could be framed as a lack of treatment for uremic syndrome and considered as supportive care at the end of life. CM finds its base in the containment of pathophysiological mechanisms related to the increase in uremic toxins and the treatment of the metabolic complications of ESRD, utilizing diet and pharmacological treatment. Conversely, palliative care intends to contain and alleviate suffering and support the best possible quality of life for patients in their final stages of life without any therapy for the underlying disease. Palliative care should not deal with diet, with the control of traditional and not traditional CV risk factors, not only because patients can experience them as unnecessary and detrimental actions at the end of life but also because the educational effort of CM requires a longer time to be effective. Distinguishing which patients should be treated by conservative management or palliative care could be a susceptible issue when the underlying disease is indolent and sneaky, such as CKD. A geriatric risk assessment of mortality over CKD could help to choose the best possible strategy for each patient.

Nephrologists have the burden of explaining the different strategies to patients and caregivers and identifying the pros and cons of both treatments. Specifically, CM is a home-based treatment, including dietary restriction, a high number of pills, the need to pursue the treatment all of the time with small but continuous changes in routine habits, a possible risk of needing RRT if urine output becomes inadequate, and the risk of unintentional weight and muscle mass loss with consequential worsening of general condition. Conversely, RRT requires 9–12 h per week for dialysis plus the time for travel with the hemodialysis option and 2–3 h per day with the peritoneal dialysis option. If RRT seems associated with a modest need for diet restriction, the higher risk of anuria could require water restriction. Finally, possible dialysis complications such as hypotension, cramps, infection, and hospitalization for vascular access or peritoneal catheter insertion should also be considered.

The treatment choice should consider the patient’s intention and the caregivers’ presence in this context. Although CM seems to require more commitment than hemodialysis to manage the patient’s diet and pharmacological treatment, it is clear that hemodialysis cannot meet all of the health needs of elderly and frail patients. Therefore, CM does not impact caregiver burden and quality of life, as reported in a recent meta-analysis [47]. Conversely, in peritoneal dialysis, caregiver effort seems greater than CM, considering the management of dialysis procedures.

## 3. Conservative Management at the Outpatient Clinic

An outpatient clinic should be organized according to the local context, but some considerations are virtually universal. Firstly, CM should be administered in the early phase of ESKD to slow the progression of CKD [28]. In case of the late referral of patients with adequate urine output, starting CM could be possible even with a lower GFR. In our experience, we usually begin CM under a 20 mL/min GFR, but this approach is also considered in all patients with permissive urine output. Protein restriction, drug support, and visit timing should be planned according to the GFR and the basal conditions. When the GFR ranges from 15 to 20 mL/min, the timing of the visit and blood examination could be every 2–3 months with protein restriction of 0.6 g/kg/day [48,49], according to general conditions, adherence to diet, and the level of awareness of treatment. For a GFR ranging from 10 to 15 mL/min, the timing of the visit and blood examination could be every 1–2 months in stable conditions [48,49], whereas protein restriction could be personalized between 0.4–0.6 g/kg/day with or without ketoanolog support, considering urea, phosphate levels, and the risk of malnutrition and sarcopenia. In patients with a GFR < 10 mL/min, we suggest a visit and blood examination at least monthly and a very low protein diet with ketoanolog support.

Secondly, a close collaboration with a dietician or dietologist is mandatory, considering that a low protein diet is a prerequisite of CM. An adequate diet allows us to have better control of the metabolic issues related to CKD [42] and theoretically positively impacts non-traditional CV risk factors such as anemia [50,51], potassium imbalance [51], and phosphate imbalance [51]. A good interaction between dietary knowledge and nephrologist skills is the best answer for ESKD to improve urea retention, calciumphosphorous metabolism, metabolic acidosis, electrolyte impairment, malnutrition, and muscle mass loss. Finally, a multidisciplinary approach should be extended to cardiologists, geriatricians, and urologists, considering the more frequent comorbidities of elderly patients with kidney impairment [52].

Thirdly, in our experience, a preliminary consultation is suggested with patients and relatives to explain the therapeutic options (RRT and CM), the meaning of the treatment, and the possible benefits and flaws of CM in all patients with a GFR < 20 mL/min. The first meeting should have an educational intent [53]. In CM, patients and caregivers should be an active part in improving care:

- Explaining the meaning of hydration, how to measure peripherical edema, how to acquire a better hydration status, or when they have to alert the nephrologist;
- Explaining the meaning of the blood examinations;
- Focusing on the treatment goals and underlining the achievement of the goal helps the patient’s trust in the CM treatment and improves their adherence to the diet and drug treatment.

## 4. Conservative Management: An Impossible Approach or a Reliable Strategy?

The need for more personnel resources, especially nephrologists [54,55], could justify the lack of a convincing CM program [56]. In the last two decades, there has been a significant reduction in nephrology fellowship and a consistently high rate of burnout among nephrologists. Parker reported in 2021 concern about the decrease in nephrology fellowships in the United States, which seems related to the complexity of kidney disease, unappealing lifestyles, and inadequate payment considering the work effort [54]. Only 25% of all age nephrologists in the United States were found to be satisfied at work, suggesting high burnout [57]. Similarly, in Europe, a survey about burnout among nephrologists showed high diffusion, considering that about 50% of participants had a high level of depersonalization and emotional exhaustion, with this being more prevalent in those working primarily in dialysis units [58].

Therefore, the statement about the lack of a CM program related to limited personnel resources seems specious when comparing the personnel employed to perform CM and hemodialysis. In CM, we assume to perform a monthly visit in older and frail patients with a GFR under 10 mL/min, which requires about 30–45 min per month for the nephrologist and nurse and about 1 h for transport to the outpatient clinic. On the contrary, for hemodialysis, the same patient requires about 2 h per month of a nephrologist’s time (considering at least 10–15 min for each hemodialysis section), at least 12 h per month of a nurse’s time (considering a 3–4 h section for three times/week), and at least 12 h for transport per month. The nephrologist and nurse’s occupancy is favorable to CM. Finally, the economic aspect also favors CM as a sustainable treatment [59,60]. In a period of cuts to healthcare system expenses, CM guarantees a sustainable and appropriate approach to clinical needs with a significant contraction of costs.

With the shortage of nephrologists, it is not improper to consider the impact of an increasing number of elderly and frail patients. CM allows for the treatment of a higher number of patients compared to hemodialysis with the same personal resources, resulting in a possible advantage for all patients, providing easy access for those who need dialysis procedures. Thus, the remarkable expansion of elderly patients who reach ESKD should induce consideration of CM as an effective therapeutic option for ESKD and the only sustainable strategy.

## 5. New Research Prospects in Conservative Management

In the last ten years, uremic toxins have become an emerging key point in defining the pathophysiological process and the clinical impact of uremic syndrome [61].

A therapeutic approach with a low-protein diet could reduce their retention and theoretically could improve the treatment of ESKD patients, reducing the morbidity and mortality of CKD. Retaining uremic toxins involves different pathological processes such as systemic inflammation, fibrosis, oxidative stress, and the impairment of cell differentiation and mitochondrial function, which impact cardiovascular damage and kidney disease progression. Uremic toxins are a heterogeneous group with different properties according to their molecular weight, solubility, and origin. The gut microbiota plays a pivotal role in the production of uremic toxins, and it is intensely affected by the amount and kind of dietary protein and fibers [62]. In addition, intestinal microbiota impairment and kidney failure amplify each other: the retention of uremic toxins due to CKD impacts badly on the intestinal epithelial barrier, increasing uremic toxins’ production, which are involved in inflammation and fibrosis processes, which further worsens kidney function [63]. Therefore, lower removal and higher production are the main mechanisms of high levels of uremic toxins in CKD patients. In the ESKD context, enhancing removal is problematic, while reducing their production seems more reasonable to achieve through dietary intervention and restoring intestinal microbial balance [64]. Developing therapeutic strategies to optimize the interaction between dietary intervention and the microbiota could be a win in the therapeutic strategy for ESKD, improving the health and prognosis of dialysis and CM patients.

So far, evidence about dietary intervention and prebiotics and probiotics’ benefits to the gut–kidney axis is insufficient to permit their applicability in clinical practice, with there being a lack of trials that prove their long-term feasibility, safety, and effectiveness [65]. Clinical trials to investigate the role of new treatment strategies for uremic toxins need considerable funding investment and research efforts, which will hopefully be supported by healthcare systems and the pharmacy industry, considering the increasing number of patients and possible profits.

In conclusion, ESKD patients have changed drastically and will change more in the future in high-income countries, with a growing prevalence of elders with high comorbidities. These patients belong to a different population in whom dialysis shows no advantage in terms of survival and quality of life. Conservative management, when possible, represents a precious option for these patients as a preliminary or definitive approach, allowing them adequate control of uremia without a significant impact on their survival and quality of life. Conservative management and palliative care are distinct kinds of care for their intent and the type of care provided to patients. Their choice should be made according to each patient’s life expectations. Finally, the outpatient clinic for conservative management should be considered a resource in the nephrology unit to care for the increasing number of frail ESKD patients.

## Data Availability

Not applicable.
